# Assessing the impact of extreme climate events on the global renewable energy market

**DOI:** 10.1016/j.isci.2025.112924

**Published:** 2025-06-18

**Authors:** Simin Shen, Lin Xiang

**Affiliations:** 1 School of Economics, Anyang Normal University, Anyang 455000, China; 2 College of Economics and Management, Zhejiang Normal University, Jinhua 321004, China

**Keywords:** Natural sciences, Climatology, Social sciences, Economics

## Abstract

Renewable energy is pivotal in addressing climate challenges, thereby requiring the systematic quantification of how extreme climate events affect market stability and resilience. Through global analysis, this study demonstrates that 74.51% of extreme climate events exert statistically significant impacts on renewable energy market. Regional analysis reveals that disasters in Asia and Europe have widespread and significant impacts on the renewable energy market, while Americas’ events elicit the swiftest market response. It also underscores the heightened significance and severity of impacts when disasters unfold in major renewable energy-producing countries. Moreover, the compounding effect of multiple climate events further amplifies their influence on the renewable energy market, with a discernible trend indicating increasingly severe consequences of climate events. Critically, this study identifies four structural determinants of renewable energy market anomalies: fundamentals, speculation, events, and climate variables, necessitating integrated frameworks to address climate-driven market uncertainties.

## Highlights

• We measured the impact of extreme climate events on the global renewable market
• The climate events in Asia and Europe are worthy of monitoring
• The increasing impacts on the renewable energy market by climate events are found
• The fundamentals, speculation, climate, and event factors should not be ignored

## Graphical abstract

**Figure:**
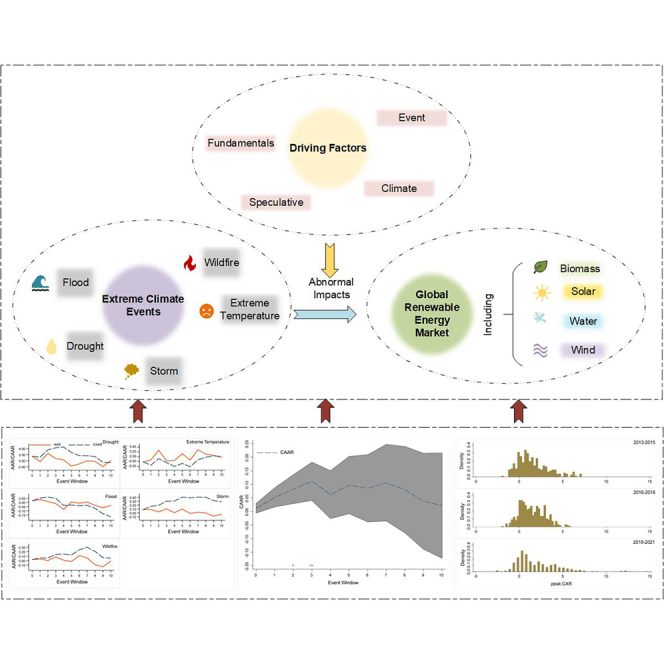

## Introduction

The escalating frequency of extreme weather events exerts substantial impacts on public health and economic productivity. Instances such as intense precipitation contribute to reduced agricultural production and a surge in commodity prices.^1^ Additionally, flooding poses a severe threat, destroying cities, assets, and, tragically, lives. With the exacerbation of climate disasters and global warming conditions, climate extremes may emerge as the preeminent global threat. As for climate mitigation actions, most countries are initiating measures to transition to less carbon-intensive practices. Renewables, including solar and wind energy, constitute critical components in climate mitigation strategies targeting the 1.5°C temperature stabilization threshold (Documents found in: https://www.iea.org/energy-system/renewables, 2023-11-23). Swift and substantial investments in renewable energies are imperative to ensure national energy security^2^ and facilitate the de-carbonization of energy systems. By enabling the nearly complete de-carbonization of electricity generation, renewables emerge as essential contributors to addressing the challenges of climate change.

Studies have widely detected the determinants of the renewable energy market, primarily focusing on demand and supply factors. These factors include fossil energy prices,^3^^,^^4^ household energy efficiency,^5^ carbon emission allowance prices,^6^ and the influence of political and economic extreme shocks.^7^ Emerging scholarship prioritizes the impact of extreme events, such as disasters,^8^^,^^9^ on energy demand and supply, given their substantial potential to induce significant natural and social disruptions. The escalating frequency of extreme climate events globally has established these phenomena as critical determinants of renewable energy system reliability and operational stability.^8^^,^^10^ This recognition underscores the necessity of incorporating considerations of extreme climate events into the broader framework of factors influencing the renewable energy market.

The intricate relationship between renewable energy production and climate disasters warrants systematic analysis. Recent studies highlight the potential of renewable energy, particularly solar energy, as a coping mechanism to mitigate electricity disruptions during disasters.^11^ However, adopting solar energy diffusion has limitations, as evidenced by adverse effects on intensive mega-solar investments.^12^ Conversely, some argue that disasters significantly and negatively impact renewable energy consumption^13^ while constraining low-carbon technology innovation.^14^ In addition, wind and solar are inherently weather-dependent, with demand and supply intricately tied to meteorological conditions. For instance, extreme temperatures can amplify cooling and heating demands,^15^^,^^16^ while also affecting hydropower^17^ and solar energy production^10^ through combined temperature and precipitation effects. Frequent climatic disasters will also lead to a rise in the demand for renewable energy and impede the progress of transitioning to cleaner energy sources.^8^ Other research has confirmed the detrimental effects of climate events on the profitability of renewable energy^18^ and the potential for climate events to impede the transition to clean energy.^19^ This multifaceted examination underscores the imperative for a nuanced understanding of how extreme climate events influence the dynamics of the renewable energy market.

Indeed, previous works have affirmed the significant influence of climate change on clean energy investment,^20^ acknowledging that extreme rainfall or temperature variations can cause notable shocks on renewable energy output to varying extents.^21^ Current scholarship predominantly examines the operability and resilience of renewable energy systems during extreme disasters.^22^^,^^23^^,^^24^ Additionally, some works explore the potential effects of extreme events on energy prices, with a predominant emphasis on fossil fuel markets (oil, natural gas) and energy stock prices,^25^^,^^26^ while also considering environmental disruptions affecting clean energy volatility.^3^ Despite these contributions, the intricate relationship between renewable energy production and weather conditions remains understudied. This research gap impedes a comprehensive analysis of how weather patterns, particularly extreme events, influence renewable energy prices. Addressing this gap is crucial for unraveling the nuanced dynamics between climate variability and renewable energy generation.

Beyond the lack of research on the consequences of climate events on the renewable energy market, methodological limitations in impact assessment persist, primarily categorized as: First, in the system simulation and evaluation model, van der Wiel et al. (2019)^27^ analyzed the meteorological sensitivity of the European renewable energy system. Perera et al. (2020)^22^ developed a stochastic, robust optimization method to consider the influence of extreme events on the Swedish renewable energy market. Ibrahim et al. (2022)^28^ applied a risk matrix method to find the possible risks of standard renewable energy systems in Malaysia during extreme climates. Zhang et al. (2019)^29^ constructed a DSGE model to quantify the disaster shock in the Chinese energy system. While these methodologies provide scientific insights into the stability and resilience of regional renewable energy systems, they commonly fall short of reflecting the determinants of global renewable energy markets. Addressing corresponding limitations is crucial for fostering a more comprehensive understanding of how global factors influence the dynamics of renewable energy markets facing extreme climate events.

Secondly, time series and econometric methods have become predominant in this research domain. Sharif et al. (2020)^30^ established the rare disasters’ predictive power over renewable energy production. Zhao et al. (2022)^14^ also revealed that disasters negatively influence energy technology innovation, revealing substantive sustainability implications. Sinha et al. (2024)^18^ applied quantile methods to detect the relationship between climate disasters and renewable energy returns. While these methodologies contribute valuable insights for quantifying the impacts of extreme climate events, further revisions are needed to elucidate the price determination mechanisms in global renewable energy markets during such events. Furthermore, it typically focuses on the broad definition of calamity without providing specific information on the diverse nature of disasters, as each type of disaster has distinct impacts. Addressing these limitations is essential for a more comprehensive understanding of the intricate pricing structure dynamics in the global renewable energy market during extreme climate events.

Besides the previously mentioned methods, empirical research has systematically applied event study frameworks to analyze the scale and dynamic effects of extreme events. This methodology has measured the impact on oil prices of various events such as wars, hurricanes^31^ and COVID-19 pandemics.^32^ Furthermore, Shen et al. (2023)^33^ utilized this method to investigate the extreme weather shocks on the global gas market. These event study applications yield critical insights into specific markets while establishing methodological frameworks for examining the impact of extreme climate events on the global renewable energy market.

From the above analysis, three critical limitations emerge in the current literature: (1) Studies predominantly focus on the regional level, such as Europe, lacking a comprehensive global perspective. Given the high frequency and complexity of extreme weather events, the current body of research falls short of determining whether these events have a generalized impact on the global renewable energy market. Although Sinha et al. (2024)^18^ advance the study of climatic extreme occurrences, their framework inadequately addresses the diversity of climate types, time periods, or other specifics. (2) Most existing studies rely heavily on engineering and systems science methods, emphasizing the enhancement of energy system controllability and disaster resilience. Nevertheless, there is a noticeable absence of in-depth discussions on the broader implications of climate events. Furthermore, prevalent single-event analyses (e.g., hurricane impacts) lack systemic integration with market dynamics. (3) There is a deficiency in the quantitative identification and analysis of the drivers behind the extreme climate shocks on renewable energy prices. Identifying these drivers is pivotal for establishing a stable renewable energy production and trading market, fostering the increased marketization of renewable energy, and facilitating the global low-carbon transition.

This article advances the field through three substantive contributions: (1) It provides a systematic global analysis framework encompassing major extreme climate events, overcoming the limitations of prior regionally constrained studies. Our global-scale investigation is conducted on a dataset consisting of 1,941 events from October 14, 2013, to December 31, 2021 to measure the specific effects of extreme climate events on the cumulative abnormal returns (CAR) of renewable energy markets (details in Table S1). This analysis provides new and detailed information on the worldwide consequences of such events. (2) It addresses the absence of heterogeneity analysis in prior work by examining many factors such as geography, time point, destructiveness, and complexity of the occurrences. This detailed research contributes to the establishment of monitoring and early warning systems, improving our comprehension of the diversity of climate occurrences. (3) The determinants of renewable energy prices during extreme events are also investigated in this article by considering fundamentals, speculation, event nature, and climate factors. This analysis provides valuable insights for decision-making to mitigate the negative impacts of extreme climate events on the renewable energy market.

## Results

### The extreme climate events’ impacts on the global renewable energy market

**Figure 1:**
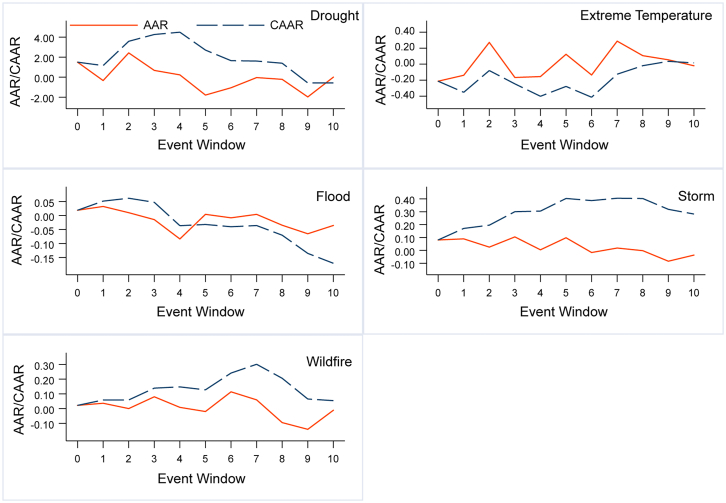
The AARs and CAARs for different disaster types Note: This figure reflects the AARs and CAARs of five extreme climate events in the event window from the day of the event occurrence to day ten.

By applying the single-sample *t*-tests for events and categories, its statistical significance identifies the proportion of significant extreme climate events for all events. The statistical significance of CARs differing from zero is assessed (Table 1), and the average abnormal return (AAR) and average cumulative abnormal return (CAAR) are illustrated in Figure 1. It is evident that droughts and extreme temperatures have a significant impact on the market on the day of occurrence, demonstrating the swift reaction of the renewable energy market to events and underscoring their substantial impact on renewable energy production. Droughts exhibit the highest ARs on day 0 and peak CARs across all samples, underscoring the catastrophic nature of this disaster. However, despite their destructiveness and persistence, extreme temperatures take the most extended number of days to reach the peak CARs, approximately nine days, which hints that extreme temperatures have a prolonged influence on the renewable market.

**Table 1:** Description of significant climate disasters

Event Type	Drought	Extreme Temperature	Flood	Storm	Wildfire	Total
Numbers of Significant	3	62	741	561	45	1412
Total Number of events	3	76	992	760	64	1895
Sig. Percent	100.00%	84.93%	74.70%	73.82%	70.31%	74.51%

The H_0_ hypothesis of *t*-test is CAR = 0, and the significant level is under 95%.

Figure 1 further illustrates that floods have the shortest effect on the renewable energy market, reaching their peak about two days after occurrence. Although floods account for about 52% of the climate events in the data samples and have notable negative impacts on renewable energy generation, particularly solar generation, they are mitigated by extensive post-disaster recovery experience and measures available to governments and residents. It leads to a reduction in their impact on the operation of renewable energy infrastructure. Additionally, some renewable energy infrastructure is often strategically located in areas less susceptible to flooding. Therefore, floods occur more frequently but have only short-term impacts due to their predictability.

Subsequently, *t*-test results (Table 1) indicate that about 74.51% of climate events significantly impact the renewable energy market. Despite their rarity, droughts have a significant impact on renewable energy production, with a 100% significance rate. Apart from droughts, temperature extremes exhibit the highest significance for the renewable energy market, at 84.93%. They not only disrupt renewable energy production, such as hydroelectric and solar power, but also increase the energy demand for cooling or heating due to the inherent nature of the disaster.

The proportion of significant flood and storm events closely follows, significantly higher than wildfire events. The lower incidence of significant wildfires can be attributed to their geographical specificity and fewer derived impacts compared to storms, floods, and temperature extremes. Additionally, wildfires are less likely to occur in core renewable energy production countries. Although 36 wildfires occurred in the main renewable energy-producing countries, with 23 of them in the United States, the main renewable energy-producing states in the US, such as Texas and Washington, experienced fewer wildfires. While wildfires did impact renewable energy production during the study period, their magnitude is relatively smaller when compared to extreme temperatures and storms, which directly affect renewable energy operations.

**Figure 2:**
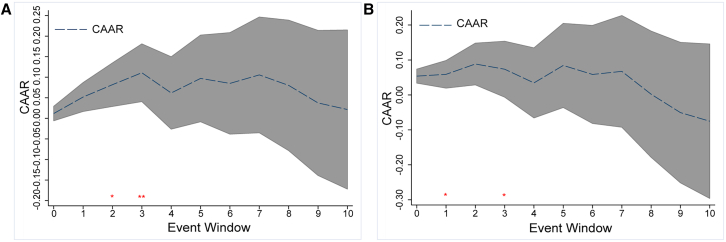
CAAR trends in event window (A) CAAR trends in the event window. (B) Robustness check of the analysis. The red ∗ and ∗∗ in the figure stand for significance levels of 5% and 10% respectively for the CAAR on the i-th day. Unless otherwise mentioned, the upper and lower intervals are set by the CAARs with the 95% confidence interval, the same as in Figures 3, 4, and 6.

The trend over time of the CAARs for all events and their significance reflected both the magnitude and duration of the consequences of climate events (Table 2, column 2; Figures 2A and 2B). The impact peaks on the third day after their occurrence and is no longer significant, hinting that the renewable energy market is susceptible to climatic events. However, the effect dissipates rapidly, likely because renewable energy currently represents a small share of the global energy market, despite the challenges posed by extreme climate conditions. As a result, the impact can be mitigated through substitution by conventional energy sources, offsetting the positive anomalous effects. It is further supported by the study of the main renewable energy-producing countries (Table 2, column 4), which reveals that the impact of a climatic event on the main renewable energy-producing countries ceases to be significant around the seventh day. This duration, which exceeds the average event duration of 6.75 days, highlights both the persistent impact of climate events and the rapid recovery capabilities of these countries.

**Table 2:** Trend in CARs due to extreme climate events

Event Window	All events	GSCI based	Main renewable energy production country	No-Main renewable energy production country	Compound Events	Non-Compound Events
	[−60,0)	[−60,0)	[−60,0)	[−60,0)	[−60,0)	[−60,0)
0	0.0118	0.0539^∗^	0.0448	0.0326	0.0687^∗∗∗^	0.0111
1	0.0519	0.0590	0.0939^∗∗^	0.0801^∗∗^	0.1115^∗∗∗^	0.0635^∗^
2	0.0819^∗^	0.0885^∗^	0.0944^∗^	0.1273^∗∗∗^	0.1150^∗∗^	0.1157^∗∗∗^
3	0.1109^∗∗^	0.0737	0.1511^∗∗^	0.1445^∗∗∗^	0.1463^∗∗^	0.1474^∗∗∗^
4	0.0618	0.0345	0.1087	0.0940	0.1006	0.0982
5	0.0972	0.0844	0.1875^∗∗^	0.1169^∗^	0.1377^∗^	0.1460^∗∗^
6	0.0851	0.0587	0.1334	0.1254^∗^	0.1460^∗^	0.1137
7	0.1057	0.0675	0.1729^∗^	0.1391^∗^	0.1738^∗∗^	0.1329
8	0.0802	0.0021	0.1624	0.1165	0.1529	0.1168
9	0.0375	−0.0505	0.0554	0.0620	0.0886	0.0358
10	0.0215	−0.0751	0.0112	0.0331	0.0364	0.0161

The CAAR in *i*-th day in the table, and ^∗^, ^∗∗^, and ^∗∗∗^ are *p*-values <0.1, *p*-values <0.05, and *p*-values <0.01, the same later in discussion.

Considering the potential missing variables, estimation errors, structural breaks, and other potential problems, this article adopts a series of robustness measures to ensure the robustness of the basic results. Details are as follows.

(1) Change windows

First, in the previous analysis in this article, the forecast window is set at 60 days before the event. To avoid the estimation errors caused by the window choice, the forecast window is adjusted to ensure the robustness of the basic results. It shortens the forecast window to 30 days and extends it to 70 days (columns 3–4 in Table S2 in supplemental information) in this part. It is evident that the results are consistent with baseline results, that the renewable energy market is susceptible to climatic events in the short term.

Second, it adjusted the event window to [0, 5], [0, 15], and [0, 30] days to observe the robustness and the persistence of disaster effects on renewable energy (column 6 in Tables S2 and S3). Consistent with the baseline results, the impact of climate disasters on the global renewable energy market is significant in the short term (2–3 days). However, if it extends the event window to 30 days, the impact can persist for up to seven days. This evidence also hints that, although climate disasters will impact the global renewable energy market, this effect will fade quickly after the event.

(2) Change variables

To check the robustness and do further broader validation, this article changes the market and global renewable energy index to estimate the climate disasters’ impacts. First, we replace the S&P 500 index with the S&P GSCI index as the market price, which contributes to a comprehensive understanding of market dynamics and is instrumental in assessing the impact of extreme climate events on the renewable energy market. Consistent with the basic results (column 5 in Table S2), the occurrence of climate events indeed brings short-term shocks to the renewable energy market.

Second, this article selects several global renewable energy indexes as the proxy for the global renewable energy market, including the iShares Global Clean Energy ETF (ICLN), Invesco Global Clean Energy ETF (PBD), VanEck Vectors Low Carbon Energy ETF (SMOG), S&P Global Clean Energy Index (S&PCE), and S&P Global Clean Energy Transition Index (GCET). These indices are frequently used as proxies for global renewable energy markets in previous literature.^34^^,^^35^^,^^36^ Table S4 provides the results for these indices; it hints that the occurrences of climate disasters undoubtedly bring significant short-term impacts on global renewable energy markets.

(3) Counterfactual check

To address the reverse causality issues resulting from investors’ precautionary behavior over potential calamities. The event window is defined as [-5, −1] and [-10, −1] for conducting counterfactual and pseudo-placebo analysis. Research demonstrates (columns 2–3 in Table S6) that investors cannot anticipate the potential impacts of extreme climate events and that there are also no reverse causality issues between the renewable energy market and the occurrence of extreme climate events.

(4) Control external factors

External shocks will also significantly impact renewable energy markets; hence, controlling other potential shocks is vital for achieving effective results. Therefore, this article takes into account the usual external shocks discussed in earlier studies^35^^,^^37^^,^^38^ and controls for several factors in the calculation of CARs, including the CBOE Volatility Index (VIX) and Crude Oil Volatility Index (OVIX), to control the investor sentiments and oil price changes’ effect on the renewable energy market. Although the VIX is a key driven factor for the climatic disasters’ impact on the renewable energy market in this article; to eliminate the investor sentiment volatilities caused by other external shocks, it is also controlled in this robustness. It also controlled Economic Policy Uncertainty (EPU) and Monetary Policy Uncertainty (MPU) to eliminate economic shocks. The results (column 6 in Table S6) are similar to the basic results that the extreme climate events bring significant impacts on the global renewable energy market in the short term.

(5) Exclude structural breaks

To avoid the potential structural break issues in the financial data, this article uses the iterated cumulative sums of squares (ICSS) method proposed by Inclan and Tiao (1994)^39^ to identify the structural breaks and exclude these breaks to do a robustness check. As depicted in Table S5 and column 5 in Table S6, the results are in line with the basics.

(6) Change models

First, this article adjusts the market model to the mean adjustment model to estimate ARs and CARs. It also hints at notable impacts (column 6 in Table S6) by extreme climate disasters within seven days, especially for the 2nd and 3rd days after the events, which is consistent with the basic results.

Second, this article reconsiders a normal fixed model in addition to the event study model to assess its robustness. The main idea behind this model is to generate a dummy variable for the event date and check its impact on CARs. Results (Table S7) show that even after controlling for the external factors, the occurrence of extreme climate events still has significant impacts on the global renewable energy market.

### Heterogeneity impacts of event occurrence location

**Figure 3:**
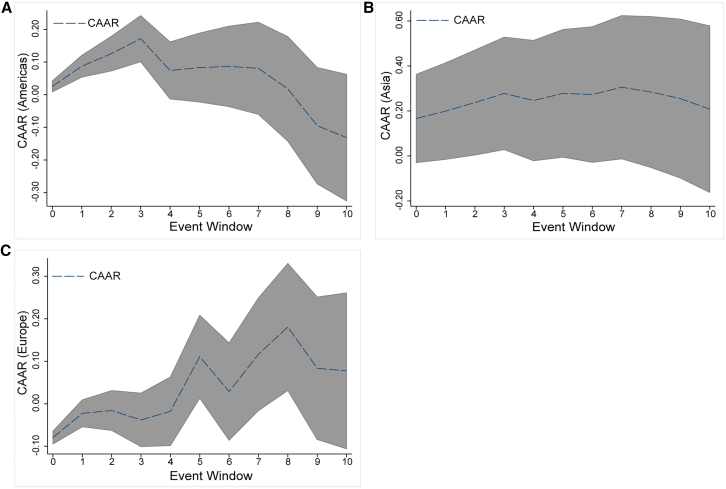
Trends in overall CAR distribution (A) Trend in the overall CAR distribution for the Americas. (B) Trend in the overall CAR distribution for Asia. (C) Trend in the overall CAR distribution for Europe. The upper and lower intervals are set by the CAARs with the 95% confidence interval.

The specific climatic conditions required for renewable energy production result in diverse production capacities specific to different geographical regions. As a result, extreme weather events in different regions tend to have heterogeneous effects on the global renewable energy market. Based on the locations of continents where extreme weather events occur, this article illustrates the overall trend in CAR for extreme weather events on each continent in Figures 3A–3C. It is clear that following extreme weather events in Asia and Europe, the renewable energy market reacted significantly and swiftly, generating substantial ARs on the day of the event in both cases. Although the trend in the impact of extreme weather events on renewable energy CARs in Asia has been relatively flat overall, it is the region with the highest impact on average CARs in the renewable energy market. Turning to Europe, the longest-lasting event influence exists in the renewable energy market, with the impacts reaching their peak CAR on the eighth day after the event. The Americas did not experience a significant impact on the renewable energy market on the day of the event. Nevertheless, it was the fastest continent to reach peak CAR and had a lower impact on overall climate extremes than other continents. This finding underscores the importance of considering regional variations in the renewable energy market’s response to climatic events.

Heterogeneity in the influence of extreme weather events can be attributed to several factors. The first is the variation in renewable energy production and adoption across regions. Asia, the largest producer of renewable energy globally, generated a substantial 3418-terawatt hours of electricity in 2021 (Date source: https://www.statista.com/statistics/610607/renewable-electricity-generation-globally-by-region/, 2023-11-23). Consequently, climate events in Asia can significantly and sustainably impact the renewable energy market. While Europe’s renewable energy production is not as high as Asia’s, the overall adoption rate of renewable energy is substantial, particularly in countries such as Sweden, where around 70% of electricity is sourced from renewable sources. Therefore, European climate disasters are inclined to have a more lasting influence on the renewable energy market. Despite the Americas also producing a considerable amount of renewable energy, the overall adoption rate is not as high, and the total production is much smaller than in Asia. As a result, disasters in the Americas often do not have as profound an influence as those in Asia and Europe.

The second reason lies in the categories of events. The Americas are most significantly affected by storms, characterized by relatively short durations, resulting in more rapid CARs than other regions’ disasters. Conversely, Asia and Europe, where extreme temperatures occur more frequently and have longer durations than floods and storms, tend to have longer events, with CAR peaks occurring over an extended period. Given that the consequences of extreme weather events are often regional^33^ and there are challenges in transporting renewable energy across regions due to limitations in renewable energy transport technologies, it is crucial to analyze further the impacts of extreme weather events on the renewable energy market in the main producing countries. This article selects ten countries—China, the United States, India, Germany, Japan, Canada, Spain, France, Italy, and Brazil—as the leading producers of renewable energy based on their installed renewable energy capacity (Renewable energy installed capacity data are from: https://www.statista.com/statistics/267233/renewable-energy-capacity-worldwide-by-country/, 2023-11-23), and conducted a detailed analysis to explore the heterogeneous location impacts.

**Figure 4:**
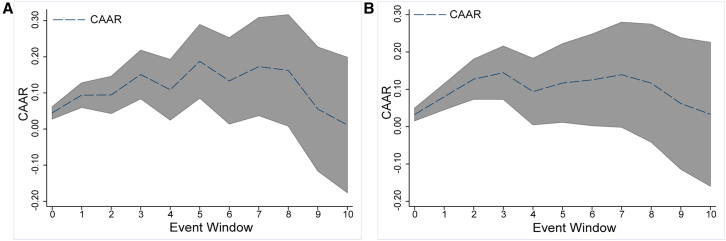
CAR distributions in main and non-main producing countries (A) Trend in the overall CAR distribution for main producing countries. (B) Trend in the overall CAR distribution for non-main producing countries. The upper and lower intervals are set by the CAARs with the 95% confidence interval.

Results (Figures 4A and 4B) indicate that extreme weather events occurring in main renewable energy-producing countries tend to have more severe impacts and peak later in the CAR than in non-main producing countries. Specifically, the peak CAR from a climate event is 0.19% in renewable energy countries and 0.14% in non-dominantly producing countries. The impact peaks on the third day after the event in non-dominant producing countries, whereas in dominant producing countries, the impact peaks on the fifth day (Table 2, columns 4–5), which suggests that main renewable energy-producing countries are more vulnerable to extreme events, and their impacts are more substantial.

It can be attributed to several factors. Despite rising energy demand associated with extreme events, it also directly affects renewable energy supply by damaging production facilities, reducing the productivity of renewable energy equipment, and, in more severe cases, leading to shutdowns. Furthermore, extreme weather events in other regions, while affecting renewable energy production to some extent and causing some cross-border transport disruptions, are mainly indirect and, therefore, less disruptive than in the main producing countries. Meanwhile, the duration of disaster impacts is slightly shorter in non-major producing countries than in major countries.

### Heterogeneity effects of extreme climate events’ damage

Highly disruptive climate events, especially those that occur near wind or hydroelectric plants, are likely to cause severe damage to renewable energy infrastructure. This damage affects the essential operation of renewable energy facilities, reduces the efficiency of energy production, and increases operating costs, ultimately impacting the global renewable energy market. Therefore, the top 50 destructive climate events and the top 50 events with the highest peak CAR for each disaster are analyzed in this article to estimate their impacts.

**Figure 5:**
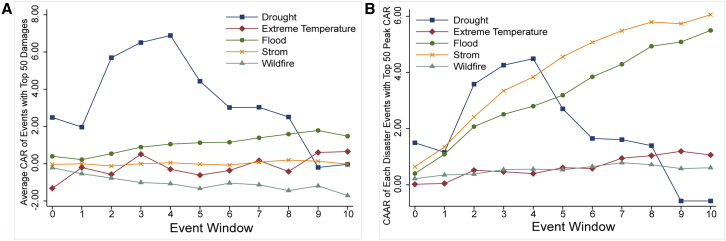
CAR trends for different damage categories (A) CAR trends for the top 50 events with the highest damage. (B) CAR trends for the top 50 disasters with the highest peak CAR.

As shown in Figure 5A of the top 50 destructive disasters, drought exhibits the highest peak CAR. Although droughts often last for over 300 days, their significant impact typically occurs within the first five days, reaching its peak on the fourth day, after which it gradually diminishes. The possible reason is that drought significantly affects water energy production in the renewable energy market, but its impact on other energy production is limited. Given their long-term sustainability and predictability, alternative energy sources are often utilized, resulting in a substantial initial impact but a reduced effect in the later stages of the disaster.

Floods and extreme temperatures are the climate events with the most severe impact on the renewable energy market after drought. There is a significant difference in how floods and extreme temperatures impact the renewable energy market. In the top 50 destructive disasters, the duration of flood events is significantly longer than that of extreme temperature events. Consequently, the impact on the renewable energy market peaks on the ninth day after floods. Meanwhile, the duration of extreme temperature events is often only about three days, resulting in a peak on day three. It suggests the need to be alert to the long-term impact of flooding on renewable energy markets and consider flood resilience when building energy infrastructure.

The overall trend of wildfires in the top 50 destructive events is negative and significant. This counterintuitive result is because while most wildfire events have a positive and significant impact, one event, August 16, 2020, in the United States, produced a substantial negative impact. This particular disaster involved several states, such as California and Washington, which are highly dependent on natural gas and traditional energy sources. Therefore, wildfires reduced the CARs value of renewable energy.

The drought trend shows no substantial change in the top 50 peak CAR events (Figure 5B). The apparent lengthening of the time it takes for extreme temperatures to peak in CAR is attributed to the significant increase in the duration (Table 3) among the top 50 peak CAR events. The time to peak has, therefore, lengthened accordingly. However, there has been a significant change in the trend of storms and floods. Despite being shorter in duration, these events take longer to reach their peak CAR, likely because, among the top 50 destructive events, storms and floods mainly occurred in areas with high climate adaptation, such as the United States. In contrast, among the top 50 peak CAR events—floods and storms— more occurred in regions with lower climate adaptation. Consequently, the impact persists even after the disaster. Wildfire events among the top 50 peak CAR events also positively impacted the renewable energy market, peaking on day seven, which indicates that wildfires can significantly impact renewable energy and transportation production and consumption. The potential impact of large-scale wildfires is particularly noteworthy due to their poor predictability.

**Table 3:** The duration of different event types (day)

Event Type	Drought	Extreme Temperature	Flood	Storm	Wildfire
TOP50 damage	364.0000	3.0000	28.0769	3.2759	109.8000
TOP50 peak	344.3333	16.3333	6.5714	3.6420	17.8367

### Characteristics of climatic events

**Figure 6:**
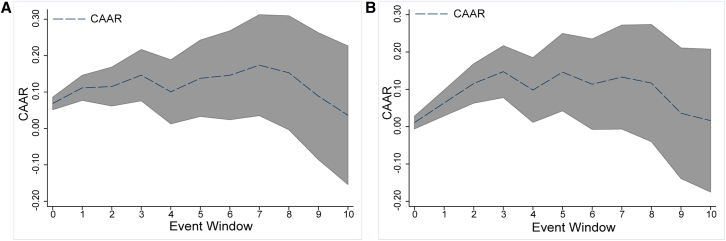
CAR distributions for varying event characteristics (A) The CAR trend for compound events. (B) The CAR trend for single events. The upper and lower intervals are set by the CAARs with the 95% confidence interval.

Extreme climate events are often interrelated: extreme temperatures may lead to drought, and severe storms may result in flooding. This article categorizes climate events into compound and single ones based on whether other sub-climate events are accompanied and discusses the impacts of these two types of events separately in this part. As observed from the general CAR trend chart (Figures 6A and 6B), whether it is a compound or a single climate event, both types result in a positive abnormal yield in the global renewable energy market on the third day after the event. It highlights the severe limitations imposed on renewable energy production and transportation processes by climatic conditions. Further analysis of the overall CAR trend reveals that the impact of compound climate events tends to be longer-lasting, with the peak of the impact often occurring on the seventh day after the event. In comparison, single climate events have a shorter duration, with the impact peaking on the third day.

The prolonged impact of compound climate events can be attributed to the overlap of multiple climate events, leading to more significant economic losses and higher medium-to short-term risks in the production process. The damage to renewable energy facilities from compound events also requires extended adjustment and maintenance times. The CAR trend also indicates that compound climate events bring more significant fluctuations in abnormal return, exerting a more substantial influence on the global renewable energy supply, underscoring the importance and urgency of preventing compound climate events for sustainable renewable energy development.

This study also considers the timing of climate events, recognizing the increasing impacts of extreme weather events in recent years due to the gradual change in climate conditions. The sample period is divided into three phases based on significant climate-related events, including the entry into force of the 2016 Paris Agreement and the COVID-19 pandemic on December 31, 2019. The CAR distribution caused by extreme weather events is then mapped to analyze the trends.

**Figure 7:**
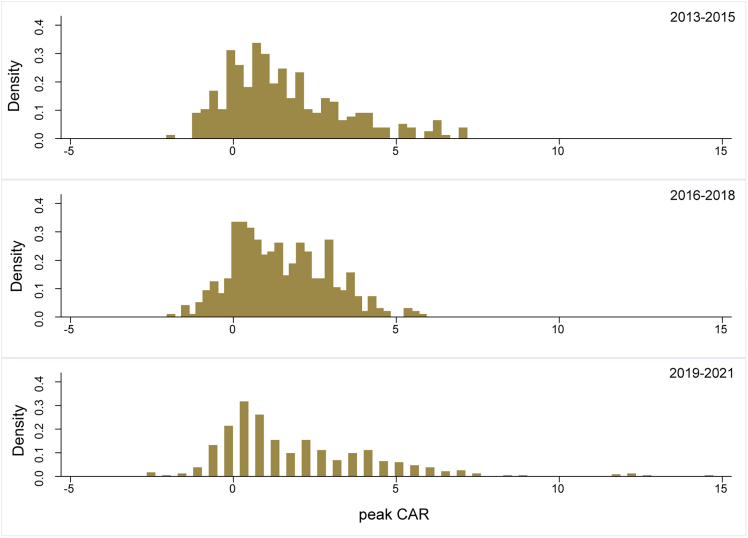
The peak CAR distribution in different periods Note: the density map of peak CAR in different periods, and the periods are marked in the upper right corner.

The density map of peak CAR (Figure 7) for each period reveals a significant right-tail distribution after 2019, with an increase in the CAR values. As extreme climate events become increasingly frequent, their impact on the renewable energy market becomes more significant. The heightened attention from governments toward climate change and increased investments in renewable energy development underscore the growing need to address the impact of extreme climate events on renewable energy production and adoption. Regarding the number of disaster events, those from 2019 to 2021 accounted for 40.38% of the total disasters, highlighting the recent increasing frequency of climate events and the urgency of preventing their tremendous impacts.

The details of peak CAR values above ten reveal that all these events occurred in May 2020, primarily in Asia, mainly in storms and floods (Table 4). It highlights the severe threats posed by storms and floods to renewable energy production under continuous climate change. These events disrupt the regular operation of renewable energy production equipment and present significant challenges to the transmission process of renewable energy. The vulnerability of disasters in Asia further emphasizes the challenges of developing renewable energy in the region. Enhancing the resilience of renewable energy production in extreme climates to improve the adaptability and stability of renewable energy equipment has become crucial for the global low-carbon transition. Addressing these challenges will contribute significantly to ensuring the reliability and effectiveness of renewable energy systems facing increasingly climate-related risks.

**Table 4:** Climate event information with peak CAR greater than ten

Disaster Start Time	Disaster Type	Occurrence Country
2020-5-15	Storm	Philippines
2020-5-17	Storm; Flood	Sri Lanka; United States of America
2020-5-20	Strom	United States of America; Bangladesh; India
2020-5-21	Flood	China
2020-5-22	Flood	Indonesia
2020-5-24	Flood	India
2020-5-25	Storm	Cuba

### Potential driving factors underlie the extreme climate events’ effects

Table 5 presents the regression results of relevant drivers influencing CARs caused by climate events. The findings offer valuable insights into the factors that can enhance the stability and resilience of renewable energy markets in the face of climate events. First, considering fundamental factors, oil and natural gas prices exhibit an apparent impact on the abnormal fluctuations under climate events, which hints that the renewable energy market is not entirely decoupled from the traditional market, influencing its pricing. Moreover, changes in oil and natural gas prices have opposite impacts, with the rise in oil prices during climate events bringing positive fluctuations to the renewable energy market, indicating that the increased cost of traditional energy might create opportunities for renewable energy as an alternative. Conversely, since gas and renewables play significant roles in the low-carbon transition, the two markets have a high degree of synchronicity. Hence, when climate events occur, the impact on the natural gas market may cause the same damage to the renewable energy market, which emphasizes the importance of improving the climate resilience of energy systems based on renewable energy and natural gas to cope with potentially extreme weather events.

**Table 5:** Driving factor regression results

Category	Indicator	(1)	(2)	(3)	(4)
Fundamentals	R_oil_	0.0153^∗∗∗^	0.0197^∗∗∗^	0.0256^∗∗∗^	0.0257^∗∗∗^
		(8.44)	(10.27)	(9.07)	(9.11)
	R_gas_	−0.0084^∗∗∗^	−0.0090^∗∗∗^	−0.0111^∗∗∗^	−0.0112^∗∗∗^
		(−5.59)	(−5.93)	(−4.97)	(−5.02)
Speculative	SVI_warm_		0.0000	0.0003^∗∗^	0.0003^∗∗^
			(0.40)	(2.37)	(2.29)
	SVI_climate_		−0.0001	0.0002	0.0002^∗^
			(−0.82)	(1.41)	(1.65)
	VIX		0.0003^∗∗∗^	0.0004^∗∗∗^	0.0003^∗∗∗^
			(7.46)	(7.35)	(7.21)
Event	Duration			−0.0001^∗^	−0.0001^∗^
				(−1.80)	(−1.84)
	Damage			−0.0000	0.0000
				(−0.02)	(0.48)
Climate	Rediness				−0.0054^∗∗^
					(−2.31)
Constant		0.0006^∗∗^	−0.0040^∗∗∗^	−0.0097^∗∗∗^	−0.0071^∗∗∗^
		(2.46)	(−2.74)	(−5.07)	(−3.14)
*N*		9141	9141	4114	4103
R^2^		0.0098	0.0167	0.0285	0.0301
Adj R^2^		0.0096	0.0162	0.0268	0.0282

*t* statistics in parentheses, and ^∗^, ^∗∗^, and ^∗∗∗^ are *p-values* < 0.1, *p-values* < 0.05, and *p-values* < 0.01.

Second, considering speculative factors, both Google’s search index (SVI) on global warming and climate change and the S&P 500 Volatility Index (VIX) positively impact renewable energy prices during extreme weather events. It suggests that, during extreme weather, investors tend to search online for information, as reflected in the increase in the SVI. The significance of this impact indicates that investors’ concerns rise regarding climate change and further influence their future investment behavior. Investors have become more attentive to renewable energy growth, impacting its market price and becoming a significant factor in investors’ decision-making, which also heightens attention and increases the events’ influence on CARs in the renewable energy market.

Moreover, the VIX significantly positively affects the renewable energy market, suggesting that investor reactions to extreme weather events have become a determining factor in the price dynamics of the market. It also indicates that energy finance is deepening in the low-carbon transition process. Renewable energy, beyond being an energy commodity, is gradually attracting the attention of speculators, acquiring significant speculative characteristics and intensifying the consequences of climate events.

The analysis of event attributes reveals that high damage from climate events does not necessarily correlate with a significant impact on the renewable energy market, at least not statistically. The possible explanation is that even though high-damage climate events result in irreversible and substantial economic losses in the local areas, these events tend to happen in countries with solid event response capabilities, such as China, the United States, and Japan. Despite causing significant property losses due to their high level of economic development, these disasters may not result in sustained harm to the renewable energy market due to robust disaster prevention and mitigation technology and response capabilities. Therefore, the high damage caused by the event does not seem to have a fatal impact on the renewable energy market. However, duration has a notable influence on the renewable energy market, and this impact decreases with the extension of the cycle of climate events. The market’s optimistic expectations for renewable energy may diminish as climate events persist, contributing to the gradual dissipation of the impacts.

The climate adaptation factor reveals a significantly negative statistical coefficient, implying that the consequence of extreme climate events on the renewable energy market is minor in countries with high climate adaptation. In other words, as countries enhance their climate resilience, the adverse effects of climate disasters on the regular operation of renewable energy systems are further reduced. This outcome can be attributed to countries with better climate resilience typically having superior renewable energy infrastructure and sufficient climate-related investments, allowing them to engage in more effective climate governance and resilience-building efforts. Consequently, these countries mitigate the negative consequences of climate shocks more effectively. On the contrary, countries with lower levels of climate adaptation exhibit less resilience when facing extreme weather events, leading to more pronounced market reactions. This result underscores the importance of investments in disaster preparedness and proactive measures for climate adaptation. While frequent extreme weather events pose serious challenges, allocating resources to climate resilience efforts enables better coping mechanisms and diminishes the negative impacts of such events on the renewable energy market.

## Discussion

Renewables emerge as essential contributors to addressing the challenges of climate change. Studies have widely detected the determinants of the renewable energy market, primarily focusing on demand and supply factors, including energy prices,^3^^,^^4^ household energy efficiency,^5^ carbon emission allowance prices,^6^ and the influence of political and economic extreme shocks.^7^ However, it is easy to ignore the extreme climate events, which are the detrimental factors that impact the profitability of renewable energy^18^ and impede the transition to clean energy.^19^ Therefore, this article considered the significant effects caused by extreme climate events on the global renewable energy market. It indicates that extreme weather events have a broad and significant influence on the renewable energy market, and their impact is gradually extending as they have become more frequent recently. Therefore, policymakers should fully recognize the potential risks brought by climate change and incorporate climate risks into the risk prevention framework. Meanwhile, it also reminds renewable energy producers to pay attention to the potential risks of extreme climate, increase production resilience, and further enhance the sustainability and stability of renewable energy supply.

Considering the current limited discussions inadequately address the diversity impacts of climate types, time periods, or other specifics^18^ on the renewable energy market. This article is especially focused on the heterogeneity analysis; it finds that the different event attributes of climate events, such as location, event type, and time point, also bring about heterogeneity of impacts on the global renewable energy market. Among them, disasters in Asia and Europe deserve further attention due to their higher impact and duration, with Asia being the continent most vulnerable to climate events. Meanwhile, the findings emphasize that events in major renewable energy-producing countries cause more significant price shocks. It can help producers adjust and deploy renewable energy layouts promptly and provide a risk warning for investors in the renewable energy market. Furthermore, it hints that no country is immune to climate change; therefore, consensus and cooperation among countries are essential for promoting renewable energy development and facilitating a low-carbon energy transition to tackle climate challenges.

Finally, the results of driving factors demonstrate the indispensability of energy fundamentals and speculative factors during extreme climate events. For the smooth operation of the renewable energy market, it is vital to continuously monitor changes in fundamental factors, form a scientific and practical risk early warning mechanism, and be alert to potential associated risks. In addition, countries should further strengthen their national capacity to adapt to climate change, guide climate investment in an orderly manner, enhance climate resilience, and reduce the negative impact of climate events.

### Conclusions

Under the macro background of frequent extreme climate events, renewable energy has become essential to deal with global climate challenges. This article analyzes the impact of climate events on the renewable energy market from five kinds of climate events. It also comprehensively analyzes the heterogeneous impacts of extreme climate events on the renewable energy market from the universality, regionality, and complexity perspectives. On this basis, the potential driving factors of the abnormal impacts from fundamentals, speculation, events, and climate factors are also considered during climate events.

The findings indicate that extreme weather events significantly impact the renewable energy market, with around 74.51% of these occurrences having a pronounced effect. Drought and extreme temperatures have the most significant impact among these events. The influence of climatic events on the global renewable energy market often manifests in the short term—within a span of seven days. The severity and trend vary based on the category, location, damage, and timing of the extreme climate event. The extensive and profound impact of disasters in Asia and Europe is remarkable, while occurrences in America provoke the most immediate market reaction. It also suggests the increased severity and intensity of consequences when disasters occur in prominent renewable energy-producing nations. Furthermore, the overlap of several climate events intensifies their effects on the renewable energy market, with a noticeable pattern reflecting the escalating severity of the repercussions of climate events recently. The impacts are driven by fundamental, speculative, event, and climatic factors. Results of this article will help further understand the price determinants and grasp the trend and law of the renewable energy market’s response to extreme climate.

### Limitations of the study

This article gives significant insights for decision-making and serves as a reference for reducing the effects of extreme climate events on the renewable energy industry. However, when discussing the impact of climate disasters, the production data will provide more precise information than the price index. Henceforth, it is possible to reevaluate the aforementioned insights using production datasets to get further insights. Moreover, we have now limited the classification of climatic disasters to five categories. However, it is imperative to include additional types of disasters in future discussions. Moreover, since the discussion of the event study method in the existing literature has not yet involved the consideration of heteroscedasticity, this article has not found a more effective way to deal with further possible heteroscedasticity. Therefore, more precise and controllable methods should be applied in the future to further explore the potential impacts of climate events.

## Resource availability

### Lead contact

Further information and requests for resources should be directed to and will be fulfilled by the Lead Contact, Lin Xiang (xianglin@zjnu.edu.cn).

### Materials availability

This study did not generate new unique reagents.

### Data and code availability

Data: The data used in this article are existing and publicly available.
Code: The original code was written in Stata and is available via the first author, Simin Shen (simin@cueb.edu.cn), upon request. The original code was written in Stata, and the sample code is stored at https://github.com/geluoxingxi/event-study. The specific codes used in this article are available via the first author, Simin Shen (simin@cueb.edu.cn), upon request.
All additional information required to reanalyze the data reported in this article is available from the lead contact upon request.

## Acknowledgments

This work was supported by the Zhejiang Provincial Philosophy and Social Science Planning Project (No. 25NDJC033YB) and Research Initiation Project for Young Teacher of 10.13039/501100004706Zhejiang Normal University (No. YS304222943).

## Author contributions

Conceptualization: S.S. and L.X.; data curation: S.S.; writing-original draft: S.S. and L.X.; writing-review and editing: S.S and L.X.; validation: S.S; funding acquisition: L.X.

## Declaration of interests

The authors declare no competing interests.

## STAR★Methods

### Key resources table

**Table:** 

REAGENT or RESOURCE	SOURCE	IDENTIFIER
**Deposited data**		

FTSE Environmental Opportunities Renewable and Alternative Energy Index	Investing	https://www.investing.com/
S&P 500 Index	Investing	https://www.investing.com/
S&P GSCI Index	Investing	https://www.investing.com/
Extreme climate events data	Emergency Events Database	https://www.emdat.be/
OPEC basket oil prices	Organization of the Petroleum Exporting Countries	https://www.opec.org/
Henry Hub gas spot price	Energy Information Administration	https://www.eia.gov/dnav/ng/hist/rngwhhdm.htm
Google trend index for global warming	Google Trends	https://trends.google.com/trends/
Google trend index for climate change	Google Trends	https://trends.google.com/trends/
CBOE Volatility Index	Investing	https://www.investing.com/
CBOE Crude Oil Volatility	Investing	https://www.investing.com/
Event Duration	Emergency Events Database	https://www.emdat.be/
Event Damage	Emergency Events Database	https://www.emdat.be/
Readiness	University of Notre Dame (NOGAIN)	https://gain.nd.edu/our-work/country-index/
Economic Policy Uncertainty	Policy Uncertainty Website	http://www.policyuncertainty.com/
Monetary Policy Uncertainty	Policy Uncertainty Website	http://www.policyuncertainty.com/
iShares Global Clean Energy ETF	Investing	https://www.investing.com/
Invesco Global Clean Energy ETF	Investing	https://www.investing.com/
VanEck Vectors Low Carbon Energy ETF	Investing	https://www.investing.com/
S&P Global Clean Energy Index	S&P Global	https://www.spglobal.com/
S&P Global Clean Energy Transition Index	S&P Global	https://www.spglobal.com/

**Software and algorithms**		

Stata 15	Stata	http://www.stata.com/

### Experimental model and study participant details

Event study model for calculating extreme events’ impact on the global renewable energy market. There are no animals, human participants, plants, microbe strains, cell lines, or primary cell cultures in this paper.

### Method details

#### Methodology

This paper employs the event study method to evaluate the responses of the renewable energy market to climate disasters. The event study approach, grounded in the efficient market hypothesis, assumes that market information is effectively incorporated into market prices,^40^ enabling scholars and market participants to assess the causal effect of an event through abnormal returns on prices.^41^ Furthermore, this method facilitates the assessment of the market impact of specific occurrences by examining abnormal stock returns within a brief period, as markets swiftly react to new information.^42^ It is a systematic and robust tool for evaluating the effects of emergency events on a specific market, extensively used in empirical studies within the finance and energy sectors.^33^^,^^43^^,^^44^^,^^45^ Since this paper intends to assess the global renewable energy market’s response to climate disasters, particularly short-term effects, for which the event study model is well-suited due to its characteristics and extensive applications.^46^^,^^47^

The abnormal returns (AR) and CAR are central to this method, which quantifies the disparities between actual and expected returns and aggregates these differences throughout the event period. The key objective of this methodology is to reveal the influence of extreme climate events on the renewable energy market. This is achieved by scrutinizing whether the ARs or CARs during the event period exhibit statistically significant deviations from zero. A substantial deviation from zero in the ARs or CARs implies a noteworthy impact of the event on the market and vice versa.

This study focuses on five categories of extreme climate events—namely, droughts, extreme temperatures, storms, floods, and wildfires—across the Americas, Asia, and Europe, comprising 1,941 discrete occurrences with a mean duration of 6.76 days (Table S1). Through calendar-based reallocation, i.e., adjusting the event date to the first open day after the event, the event timetable was temporally aligned with financial market operations. Methodological specifics include.

#### Calculation of ARs and CARs

Before calculating ARs and CARs, the events are defined in this paper. The climate disaster data derive from the EM-DAT database, with the disaster start date designated as the event date. Subsequently, AR and CAR are computed across event timelines with statistical significance testing. The meticulous definition and analysis of events ensure a robust examination of the impact of extreme climate events on the renewable energy market.

For the calculation of expected returns, various models from the existing literature have been considered, such as the market model,^33^^,^^48^ the Fama-French model,^49^^,^^50^ and autoregressive models.^51^ Aligning with the prevailing practice in the field, this study chooses the market model, widely recognized as the most commonly employed model among these alternatives, to estimate expected returns. The S&P 500 is selected as the market index in this paper as it serves as the benchmark for global equity markets given its representative market capitalization. Furthermore, the estimate window in this paper is defined as 60 days before the event, denoted as the estimate window [-60, 0). The event windows are set at [0, 10] and [0, 5] for fundamental analysis and robustness checks, primarily focusing on short-term impacts. The specific formula for this calculation is as follows:

(Equation 1) $$R_{it}=\alpha_{i}+\beta_{i}R_{mt}+\varepsilon_{it}$$

(Equation 2) $$E\left[R_{it\left(event\right)}\right]=\alpha_{i}+\beta_{i}R_{mt\left(event\right)}$$

(Equation 3) $$AR_{it}=R_{it\left(event\right)}-E\left[R_{it\left(event\right)}\right]$$

Where $AR_{it}$ is the AR of event *i* at time *t*. $R_{it\left(event\right)}$ is the log renewable energy return at time *t*, and $E\left[R_{it\left(event\right)}\right]$ is the expected return, assuming that the event does not occur.

#### Significance tests of event shock

Two empirical validation procedures are established: First, we examine whether CARs within the [0,10] event window demonstrate statistical significance, identifying the proportion of significant extreme climate events for all events. Second, we assess cross-sectional variation through mean CARs significance testing for all events and for events in different geographic regions. The details are formulated as follows:

(Equation 4) $$CAR_{it}=\sum_{i=0}^{10}AR_{it}$$

(Equation 5) $$t_{1}=\frac{CAR_{it}}{VAR\left(CAR_{it}\right)/\sqrt{n}}$$

Where $CAR_{it}$ is the CARs of event *i* in time *t*.

The subsequent analysis involved a daily test to ascertain whether the mean CARs (CAARs) of all events or all events of the same region exhibited a significant departure from zero. The construction of the *t*-test statistic for this evaluation is illustrated as follows:

(Equation 6) $$t_{2}=\frac{\bar{CAR_{it}}}{\sqrt{VAR\left(\bar{CAR_{it}}\right)}}$$

$\bar{CAR_{it}}$ is the mean of $CAR_{it}$ for all events or all events in a certain region, it is usually denoted as CAAR in the analysis.

#### Potential driving factor analysis

After checking the extreme climate events’ significant impacts, the potential driving factor is also investigated in this paper by the following setting:

(Equation 7) $$CAR_{i}=f\left({\text{fundimental}}_{i},{\text{speculative}}_{i},{\text{event}}_{i},c\;\lim\;{\text{ate}}_{i}\right)$$

Here, the subscript *i* represents the *i*-th event. The driving factors encompass four parts: fundamental, speculative, event and climate factors, details in Table 1.

The selection of driving factors is primarily based on previous studies.^33^ The specific reasons are as follows: First, fundamental factors revolve around substitution effects among energy markets during catastrophic climate events. Given that oil and gas are formidable substitutes for renewable energy,^52^ substitution across various energy markets following a disaster is likely to drive impacts for the renewable energy market in the aftermath of a climate event. Second, the choice of speculative factors primarily relies on investor sentiment; catastrophic climatic disasters and ensuing emergency responses frequently provoke public concern. Moreover, individuals actively pursue disaster-related and climate change information via search engines or social media before and following an event, thereby affecting investor expectations and adjusting investment portfolios across markets.^53^ Therefore, speculative factors could also be potential driving forces for climate disasters’ impacts on the renewable energy market. Third, regarding event factors, the considerable heterogeneity of the effects of climate disasters with varying damages and durations on the renewable energy market^33^ should be acknowledged as a valid driver within the research framework. Finally, the climate factor indicates that the influence of climate events on the renewable energy market varies according to countries’ levels of climate preparedness^45^; hence, it may also be regarded as a significant driver.

**Table:** Details of driving factors

Catergory	Indicator	Description or calculation method
Fundimental	R_oil_	The log return of OPEC basket oil prices
	R_gas_	The log return of Henry Hub gas spot price
Speculative	SVI_warm_	Google trend index for global warming
	SVI_climate_	Google Trend Index for Climate change
	VIX	S&P 500 volatility index
Event	Damage	Event damage discounted by CPI
	Duration	Event duration
Climate	Readiness	Climate readiness for a country in event year

Note: Rediness is an indicator developed by the University of Notre Dame.^33^^,^^45^

#### Variables

This study employs the FTSE Environmental Opportunities Renewable and Alternative Energy Index as the renewable energy market data, obtained from the Investing website (https://www.investing.com/). As a constituent of the FTSE Environmental Opportunities Index Series, this index measures the performance of global companies significantly involved in environmental business activities, which is widely utilized in sustainability and renewable energy research.^54^^,^^55^^,^^56^ For additional market data, the S&P 500 and S&P GSCI indexes are sourced from the Investing website and S&P Global website, respectively, enabling a comprehensive understanding of market dynamics and are instrumental in assessing the impact of extreme climate events on the renewable energy market. The driving factors utilized in this study integrate multi-data streams from OPEC, EIA, Google Trends, NDGAIN, and the Emergency Events Database (EM-DAT) spanning a sample period from October 14, 2013, to December 31, 2021 (details in Details of driving factors). After excluding events with an estimation window of fewer than 60 days, the conclusive dataset comprises a total of 1941 events. These events are categorized into 1011 floods, 785 storms, 77 extreme temperatures, 65 wildfires, and three droughts; detailed disaster information is provided in Table S1.

As depicted in Table S1, floods and storms account for 92.5% of all extreme climate events, highlighting their predominant role. These two categories are particularly significant due to their potential to disrupt the regular operation of solar and wind power systems (Brás et al., 2023)^57^ highlighting the critical need to assess their impacts on the renewable energy market. Despite their lower frequency, droughts are included in this study due to their prolonged duration and substantial impact, including the highest level of damage. Extreme temperatures, which rank second only to drought in terms of damage, pose a significant threat in Europe, impacting plants, crops, and critical infrastructure, leading to elevated immediate and subsequent costs. Furthermore, extreme temperatures threaten renewable energy production, including hydropower, and contribute to higher consumer costs. The multifaceted consequences of extreme temperatures underscore their profound implications for the environment and the economic landscape.

An analysis of specific continents reveals that Asia experiences the highest frequency of extreme weather events, nearly matching the combined occurrences of the other two continents. Within Asia, floods and storms are the most prevalent events, with shorter durations. In comparison, the Americas report fewer extreme weather events than Asia, yet the associated damages surpass those of Asia and Europe. Notably, the Americas also experience the highest frequency and longest average duration of wildfires. In contrast, Europe faces distinctive challenges, notably affected by extreme temperatures, resulting in severe and prolonged impacts. These challenges are compounded by energy shortages, setting Europe apart with unique challenges posed by extreme temperature events. The varying patterns across continents underscore the need for nuanced approaches to understanding and addressing the impacts on renewable energy markets.

### Quantification and statistical analysis

#### The proportion of significant extreme climate events for all events

Initially, we computed the *t*_*1*_ statistics for all extreme climate events utilizing Equations 4 and 5, categorized these statistics by disaster type, determined the proportion of significant *t*_*1*_ statistics, derived the significant proportions of various climate extreme events, and compiled the results into Table 1. Our analysis reveals that 74.51% of extreme climatic events significantly affect the global renewable energy market at a 95% significance level, with 100% of droughts, 84.93% of extreme temperatures, 74.70% of floods, 73.82% of storms, and 70.31% of wildfires demonstrating considerable impacts.

#### The extreme climate events’ impacts on global renewable energy market

We use the Equation 6 to calculate the significance of abnormal returns in event windows and plot the results in Stata. The results indicate that the extreme climate event significantly affects the global renewable energy market on the second and third days, with CAAR levels of 0.0819 and 0.1109 corresponding to significance levels of 90% and 95%, respectively. Therefore, we conclude that the effects caused by extreme climate events are generally short-term. Additionally, we used the same method to conduct several robustness checks, and the details are provided in the Tables S2–S7. To provide location heterogeneity, we screened out the events that occurred in the Americas, Asia, and Europe based on the locations of continents in the EM-DAT database. Similarly, we used Equation 6 to test the significance of extreme climate events and utilized the Stata tool for visualization.

Besides, we manually cleaned out the data from major renewable energy-producing countries and non-major renewable energy-producing countries and presented results in the paper by the same means. We find that the extreme climate events that occur in major renewable energy countries have significant impacts from day one to day seven, except for day four and day six. The CAARs are 0.0939 (significant at the 95% significance level), 0.0944 (significant at the 90% significance level), 0.1511 (significant at the 95% significance level), 0.1875 (significant at the 95% significance level), and 0.1729 (significant at the 90% significance level). Disaster occurrences in non-main countries are significant from day one to day seven except for day four. The CAARs are 0.0801(under 95% significant level), 0.1273(under 99% significant level), 0.1445(under 99% significant level), 0.1169(under 90% significant level), 0.1254 (under 90% significant level), and 0.1391(under 90% significant level). It hints that the peak CAR from a climate event is 0.19% in dominant renewable energy countries and 0.14% in non-dominantly producing countries. The impact peaks on the third day after the event in non-dominant producing countries, whereas in dominant producing countries, the impact peaks on the fifth day (Table 2, columns 4–5), which suggests that main renewable energy-producing countries are more vulnerable to extreme events, and their impacts are more substantial.

Moreover, we categories climate events into compound and single ones based on whether other sub-climate events are accompanied and discuss the impacts of these two types of events separately. The results show that when compound events occur, they are more likely to bring more significant and long-lasting impacts on renewable energy markets. Its CAARs are significant from day zero to day seven except for day four. The CAARs are 0.0687 (99%), 0.1115 (99%), 0.1150 (95%), 0.1463 (95%), 0.1377 (90%), 0.1460 (90%), and 0.1738 (95%), respectively (the values in parentheses are the levels of significance, the same as below). However, for the non-compound extreme climate events, it only significantly impacts the global renewable energy market from day one to day three and day five, with the CAARs of 0.0635 (90%), 0.1157 (99%), 0.1474 (99%), and 0.1460 (95%).

#### Analysis of potential driving factors underling the extreme climate events’ effects

In this part, we use the OLS regression model to recognize the potential driving factors, the results show that the driving factors are certainly impacts the relationships between extreme climate events and the global renewable energy market. The model including eight factors has the F(8, 4094) = 15.90 and R^2^ = 0.0301, with an adjusted R-squared of 0.0282. Specific for factors, oil (coefficient is 0.0257, *p* = 0.000) and natural gas (coefficient is −0.0112, *p* = 0.000) prices exhibit an apparent impact on the abnormal fluctuations under climate events, which hints that the renewable energy market is not entirely decoupled from the traditional market, influencing its pricing.

Both Google’s search index (SVI) on global warming (coefficient is 0.0003, *p* = 0.022) and climate change (coefficient is 0.0002, *p* = 0.100) and the S&P 500 Volatility Index (VIX) (coefficient is 0.0003, *p* = 0.000) positively impact renewable energy prices during extreme weather events. The analysis of event attributes reveals that high damage from climate events does not necessarily correlate with a significant impact on the renewable energy market, at least not statistically. However, duration has a notable influence (coefficient is −0.0001, *p* = 0.065) on the renewable energy market, and this impact decreases with the extension of the cycle of climate events. The market’s optimistic expectations for renewable energy may diminish as climate events persist, contributing to the gradual dissipation of the impacts. The analysis of the climate adaptation factor reveals a significantly negative statistical coefficient (coefficient is −0.0054, *p* = 0.021), implying that the consequence of extreme climate events on the renewable energy market is minor in countries with high climate adaptation.

### Additional resources

All data and code resources are provided in the key resources table and resource availability sections. More potential information will be fulfilled by the lead contact.
