# Supplementary material for: Assessing the impact of extreme climate events on the global renewable energy market
Source: iScience. 2025 Jun 18;28(7):112924. doi: 10.1016/j.isci.2025.112924 (PMC12274797; doi:10.1016/j.isci.2025.112924)
Supplement: Document S1. Tables S1–S7 [file mmc1.pdf]

iScience, Volume *28*

Supplemental information

Assessing the impact of extreme climate events on the global renewable energy market

Simin Shen and Lin Xiang

Supplemental information

Data S1

Table S1 Related to STAR Methods

Table S1. Descriptive statistics of events

Region	Events Number	Event Type	Number of which	Average Damage (1000 $)	Average Duration (day)
Americas	615	Drought	2	6352534.00	334.50
		Extreme temperature	8	1197674.00	27.43
		Flood	298	741209.90	7.34
		Storm	274	3402654.00	4.19
		Wildfire	33	3050056.00	29.55
Asia	996	Drought	1	3348087.00	364.00
		Extreme temperature	28	723080.00	13.85
		Flood	564	988650.80	7.90
		Storm	391	937124.90	2.30
		Wildfire	12	311797.70	20.83
Europe	330	Drought	0	0.00	0.00
		Extreme temperature	41	6048157.00	17.44
		Flood	149	1253734.00	4.03
		Storm	120	470890.80	1.45
		Wildfire	20	278316.10	4.11

Note: In the subsequent analysis, the reduction in the number of extreme events was due to the elimination of some events with insufficient estimation windows.

Table S2-S7 Related to Results

Table S2. Robustness results (basic)

Event Window	All events	Change Window	Change Window	GSCI based	Change Predict Window
	[-60, 0)	[-30, 0)	[-70, -10)	[-60, 0)	[-60, 0)
0	0.0118	0.0178	0.0123	0.0539*	0.0061
1	0.0519	0.0482	0.0453	0.0590	0.0437
2	0.0819*	0.0842**	0.0774*	0.0885*	0.0737*
3	0.1109**	0.0973*	0.1041**	0.0737	0.1018**
4	0.0618	0.0492	0.0552	0.0345	0.0493
5	0.0972	0.0777	0.0837	0.0844	0.0817
6	0.0851	0.0642	0.0700	0.0587	
7	0.1057	0.0841	0.0919	0.0675	
8	0.0802	0.0535	0.0577	0.0021	
9	0.0375	0.0127	0.0153	-0.0505	
10	0.0215	-0.0093	-0.0047	-0.0751	

Note: the CAARs on the *i*-th day in the table and *, **, and *** are p-values for *t*-tests, and $p < 0.1$, $p < 0.05$, and $p < 0.01$ are the same below.

Table S3. Extend the event window

Event Window	All events	All events
0	0.0137	0.0131
1	0.0519	0.0512
2	0.0884**	0.0885**
3	0.1193**	0.1263**
4	0.0700	0.0790
5	0.1063*	0.1164*
6	0.0992	0.1096
7	0.1220	0.1374*
8	0.0989	0.1177
9	0.0569	0.0806
10	0.0417	0.0704
11	0.0653	0.0968
12	0.0675	0.0994
13	0.0339	0.0700
14	0.0376	0.0782
15	0.0649	0 .1127
16		0.1557
17		0.1487
18		0.1155
19		0.0961
20		0.0973
21		0.1363

Event Window	All events	All events
22		0.1475
23		0.0904
24		0.0720
25		0.1110
26		0.1899
27		0.1765
28		0.1804
29		0.2079
30		0.2443

Table S4. Robustness results of change variables

Event Window	ICLN	SMOG	PBD	GCET	S&PCE
Predict window	[-60,0)	[-60,0)	[-60,0)	[-60,0)	[-60,0)
0	0.0403	0.0299	0.0313	0.0430	0.0792**
1	0.0737	0.0739*	0.0792*	0.0975*	0.0739
2	0.1082*	0.0659*	0.0807*	0.0996*	0.0848*
3	0.0894	0.0392	0.0840	0.1149*	0.0297
4	0.0550	0.0221	0.0145	0.0439	-0.0241
5	0.0956	0.1143	0.0824	0.0860	0.0585
6	0.0881	0.1249	0.0897	0.0891	0.0449
7	0.1061	0.1159	0.1061	0.0749	0.0229
8	0.0354	0.1052	0.0555	0.0060	-0.0878
9	-0.0037	0.0532	-0.0074	-0.0659	-0.1540
10	0.0109	0.1467	0.0265	-0.0299	-0.1199

Table S5. The structural breaks in renewable energy market

Date	Return
2015-05-20	-0.0199
2015-10-07	0.0079
2016-01-04	-0.0253
2016-04-06	-0.0042
2016-06-10	-0.0266
2016-07-06	-0.0118
2016-12-16	-0.0025
2020-02-24	-0.0298
2020-04-02	0.0057
2021-03-12	-0.0056

Table S6. Counterfactual check, exclude structural breaks and external factors, and change model results

Event Window	All events	All events	Event Window	No breaks	Control external factors	Change estimate model
Predict window	[-60, -5)	[-60, -10)	Predict window	[-60, 0)	[-60, 0)	[-60, 0)
0			0	0.0074	0.0216	0.0543*
-1	0.0600	0.0734	1	0.0456	0.0558	0.0907**
-2	0.0757	0.0844	2	0.0737*	0.0919*	0.1419**
-3	0.0241	0.0343	3	0.1088**	0.1332*	0.1328**
-4	0.0237	0.0351	4	0.0537	0.1289	0.1210
-5	-0.0125	0.0023	5	0.0928	0.2281	0.1667**
-6		0.0118	6	0.0780	0.2734	0.1396
-7		0.0223	7	0.0944	0.3408	0.1712*
-8		0.0106	8	0.0799	0.3844	0.1040
-9		0.0085	9	0.0233	0.5134	0.0597
-10		0.0151	10	0.0074	0.7091*	0.0258

Note: Columns 2-3 in this table is the result of that assumption: the event window is the days before the event—5 days and 10 days.

Table S7. Robustness results for change models

Variables	(1)	(2)	(3)
Event	0.0009*	0.0012***	0.0008*
	(1.88)	(2.64)	(1.82)
Ln_EPU			0.0089***
			(4.72)
Ln_MPU			0.0015*
			(1.91)
Ln_VIX			-0.0209***
			(-15.71)
Ln_OVIX			0.0063***
			(5.10)
Constant	0.0001	-0.0000	-0.0170**
	(0.41)	(-0.11)	(-2.07)
N	9141	9141	9141
Time Fixed Effects	No	Yes	Yes
R^2	0.0004	0.0175	0.0457
adj. R^2	0.0003	0.0165	0.0444

Note: *t* statistics in parentheses, and *, **, and *** are $p < 0.1$, $p < 0.05$, and $p < 0.01$.
